# Good Death and Quality of End-of-Life Care in Patients with Coexisting Cancer and Dementia: Perspective of Bereaved Families

**DOI:** 10.1089/pmr.2023.0083

**Published:** 2024-07-13

**Authors:** Ayumi Takao, Harue Arao, Sena Yamamoto, Miwa Aoki, Katsuyasu Kouda, Tatsuya Morita, Yoshiyuki Kizawa, Satoru Tsuneto, Yasuo Shima, Kento Masukawa, Mitsunori Miyashita

**Affiliations:** ^1^ Graduate School of Nursing, Osaka Metropolitan University Graduate School of Nursing, Osaka, Japan.; ^2^ Division of Health Sciences, Osaka University Graduate School of Medicine, Suita, Japan.; ^3^ Department of Hygiene and Public Health, Kansai Medical University, Hirakata, Japan.; ^4^ Department of Palliative and Supportive Care, Palliative Care Team, Seirei Mikatahara General Hospital, Hamamatsu, Japan.; ^5^ Department of Palliative and Supportive Care, Institute of Medicine, University of Tsukuba, Tsukuba, Japan.; ^6^ Department of Human Health Sciences, Kyoto University Graduate School of Medicine, Kyoto, Japan.; ^7^ Department of Palliative Medicine, Tsukuba Medical Center Hospital, Tsukuba, Japan.; ^8^ Department of Palliative Nursing, Health Sciences, Tohoku University Graduate School of Medicine, Sendai, Japan.

**Keywords:** complicated grief, dementia, good death, quality of end-of-life care, terminal cancer

## Abstract

**Background:**

Patients with coexisting cancer and dementia often have complex health care needs and face challenges in achieving a good death.

**Objectives:**

To evaluate good death achievement and end-of-life (EOL) care in patients with coexisting cancer and dementia from the perspective of bereaved families.

**Design:**

Cross-sectional nationwide postal survey.

**Setting/Subjects:**

Bereaved families of patients with cancer who died in hospice and palliative care units across Japan.

**Measurements:**

Bereaved families completed an anonymous, self-reported questionnaire. Their perspective on achieving a good death was assessed using the Good Death Inventory (GDI) (total score: 18–126). The Revised Care Evaluation Scale—short version (CES2) was used to assess EOL care (total score: 10–60). We examined the Brief Grief Questionnaire (BGQ) (total score: 0–10) and Patient Health Questionnaire 9 (PHQ9) (total score: 0–27).

**Results:**

Data from 670 participants were analyzed, including 83 (12.4%) bereaved families of patients with coexisting cancer and dementia. No statistical differences were observed in the total GDI score for 18 items (dementia comorbidity vs. nondementia comorbidity groups, mean ± standard deviation, respectively, 78.4 ± 17.7 vs. 80.0 ± 15.5, adjusted [adj] *P* = 0.186), CES2 score (49.70 ± 9.22 vs. 48.82 ± 8.40, adj *P* = 0.316), BGQ score (3.40 ± 2.41 vs. 4.36 ± 2.28, adj *P* = 0.060), and PHQ9 score (4.67 ± 4.71 vs. 5.50 ± 5.37, adj *P* = 0.788).

**Conclusions:**

GDI, CES2, BGQ, and PHQ9 scores did not differ significantly between groups, regardless of the presence of dementia in hospice and palliative care units. Patients with coexisting cancer and dementia can achieve a good death by high-quality EOL care.

## Key Message

Providing high-quality end-of-life care for patients with coexisting cancer and dementia and their families so that they can achieve a good death.

## Introduction

Older patients with cancer commonly have comorbid conditions.^1^ As the global population ages, the proportion of patients with coexisting cancer and dementia is increasing.^2^ In hospices in the 1990s, approximately 7% of patients had a terminal illness and comorbid dementia,^3^ compared with approximately 30% now.^4^ Therefore, providing high-quality end-of-life (EOL) care for patients with coexisting cancer and dementia to facilitate good and peaceful death is important.

Patients with coexisting cancer and dementia often have complex health care needs such as a high prevalence of insomnia, neuropsychiatric symptoms, low opioid use, and possibly inadequate symptom control.^5–7^ In addition, they exhibit higher rates of hospitalization, emergency department visits, and readmission than patients with cancer and without dementia.,^8^ However, low access to specialized palliative care remains a clinical challenge.^9,10^ Patients with dementia may not have access to tailored or better care, and a holistic perspective could be lacking when dementia coexists with other terminal illnesses.^11,12^

Achieving a good death is the ultimate goal of hospice care, benefiting both the patients and their families. Good death is defined as a death “free from avoidable distress and suffering for patients, families, and caregivers; in general accord with patients’ and families’ wishes; and reasonably consistent with clinical, cultural, and ethical standards.”^13^ This concept focuses on how to best live if death is inevitable.^14–16^ Achieving a good death, even if a decline in physical function is inevitable at the end of life, preserves the patient’s comfort and dignity^17–19^ and can improve overall affirmation of the patient’s life. This can also palliate families’ anxiety and fears surrounding the loss of a loved one.^15^ In fact, lower rates of complicated grief have been reported when the family perceives the attainment of a good death.^20,21^

Achieving a good death is associated with the quality of EOL care, that is, care coordination among hospice staff^22^ and timely referral to palliative care units.^23^ Therefore, patients with coexisting cancer and dementia may not achieve a good death. The quality of death in patients with coexisting cancer and dementia has been investigated from the perspective of health care providers, reporting lower scores for patients with dementia.^24^ However, similar studies have not been sufficiently conducted from the perspective of the families of such patients.

This study aimed to (1) compare the achievement of a good death, EOL care structure and process, and support for the families of patients with coexisting cancer and dementia who die in hospice and palliative care units and patients with cancer who do not have dementia and (2) compare the degree of complicated grief and depression of bereaved families between patients with coexisting cancer and dementia and patients with cancer who do not have dementia.

## Methods

This research was a part of the Japan Hospice and Palliative Care Evaluation Study (J-HOPE) 4—a national study program that evaluated EOL care. We sent a cross-sectional, anonymous questionnaire between July and September 2018 to bereaved families of patients with cancer who died in hospice and palliative care units. The details of the research program are presented in the protocol article.^25^

### Participants

The participants were bereaved families of patients with cancer who died in hospice and palliative care units in Japan. The study comprised 184 hospice and palliative care units; 80 bereaved families who met the selection criteria were retrospectively identified. The inclusion criteria were (1) bereaved family members aged ≥20 years, (2) families of patients who died from cancer, and (3) patients aged ≥20 years. The exclusion criteria were (1) patient stay in palliative care setting <3 days, (2) family contact information could not be obtained, (3) patient death in an intensive care unit or treatment-related death, (4) family experienced severe psychological distress, and (5) families incapable of completing the self-reported questionnaire owing to visual disability or cognitive decline.

Bereaved families responded to questions pertaining to the dementia diagnosis and cognitive status of the patients. Dementia comorbidity groups have been defined in a previous study reporting the caregiver burden for families of patients with coexisting cancer and dementia.^26^ If the respondents answered that the patients were diagnosed with dementia, patients were classified in the dementia comorbidity group. Informing patients and their families of a dementia diagnosis is common in Japan.^27,28^ Therefore, if the respondents indicated that the patients were diagnosed with dementia, patients were classified in the dementia comorbidity group. If the patients were not diagnosed with dementia and had no cognitive impairment based on the statement of the bereaved families, they were classified in the nondementia comorbidity group.

### Procedures

Questionnaires were sent directly from the participating institutions to the bereaved families by mail. The patients’ primary family caregivers were instructed to provide responses to the questionnaire and send it back to the secretariat. If they did not wish to answer, they were asked to check the “no participation” box and return the unanswered questionnaire. A reminder was sent to the families one month after sending the questionnaire. A pilot test was conducted with the questionnaire before the study. The face validity of six bereaved families was assessed. This research program was approved by the institutional review boards of Tohoku university hospital and participating institutions.

### Measurements

#### Good death inventory: short version

The degree of achieving a good death was evaluated using the Good Death Inventory (GDI) from the bereaved family’s perspective.^29^ The original version of the GDI includes 18 domains (10 core and 8 additional domains) and 54 items. The core domains assess attributes that most people consider vital, and the additional domains assess attributes that are vital depending on individual values. The reliability and validity of the GDI-short version, which comprises 18 representative items in each domain, have been established.^29^ Participants scored each item from 1 (absolutely disagree) to 7 (absolutely agree). The scores for all items, core domain items, additional domain items, and overall evaluation of achieving a good death were calculated to determine the final score. A high score implied achieving a good death. The GDI is currently used in Western and Eastern countries^30,31^ and has been continuously adopted in Japan in the 2007,^32^ 2010, 2014,^33^ and 2018^25^ J-HOPE studies.

#### Revised care evaluation scale: Short version

The Revised Care Evaluation Scale (CES2) comprising 10 domains and 28 attributes^34^ is used to quantify the quality of EOL care with a focus on the process and structure of EOL care provision. Participants scored each item from 1 (highly disagree) to 6 (highly agree). The short version of the CES2 includes 10 representative items in each domain and was used in this study.^34^ Higher scores indicated high-quality EOL care. The reliability and validity of this scale have been confirmed.^34^

#### Support for families by health care professionals

To evaluate the support families received from health care professionals, we developed seven questions pertaining to the care provided in the palliative care unit. Participants were asked to quantify the received support from 1 (strongly disagree) to 4 (absolutely agree). Owing to the lack of validated measurement tools, the questions were created in combination with findings from previous studies,^35,36^ discussions between authors, and the pilot study results.

#### Brief grief and depression

To assess complicated grief, the Brief Grief Questionnaire (BGQ) was used.^37^ This measurement has been used in Japanese individuals, including bereaved families whose loved ones died from cancer. The items were scored on a 3-point Likert scale (0–2). If the total score was ≥8, the respondents were considered to have complicated grief. To assess depressive symptoms, the Patient Health Questionnaire 9 (PHQ9) was used.^38^ The items were scored on a 4-point Likert scale (0–3). The respondents were considered to have mild or high depression if the total score was ≥10. The reliability and validity of the Japanese version of these questionnaires have been confirmed.^39,40^

#### Demographics

Data pertaining to patient age, sex, duration of stay in the palliative care unit, duration of anticancer treatment, care level, monthly medical expenses, and cancer type were collected. In addition, data pertaining to the bereaved family member’s sex, relationship with the patient, age, and number of days since death were also assessed. The patient’s age, sex, cancer type, and duration of stay in the palliative care unit were provided from medical records by the person in charge at the participating facilities. The family members provided all additional information on the questionnaire.

### Statistical analysis

Based on the presence of dementia, bereaved families were categorized into two groups. We calculated descriptive statistics for the measured variables. For categorical and continuous variables, chi-square and Student’s *t*-tests were performed, respectively. In addition, Student’s *t*-tests were used to compare the scores for all items and total scores on the GDI and CES2 and for all items assessing support for families by health care professionals between the two groups. We also calculated Cohen’s *d* to determine the effect size (ES). The Student’s *t-*test was also used to compare the total BGQ and PHQ9 scores. Age-adjusted (adj) *P* values were calculated for each item using an analysis of covariance. Statistical significance was considered at a two-tailed *P* < 0.05. All analyses were performed using IBM SPSS Statistics for Windows, version 28.0 (IBM, Tokyo, Japan).

## Results

**FIG. 1.**
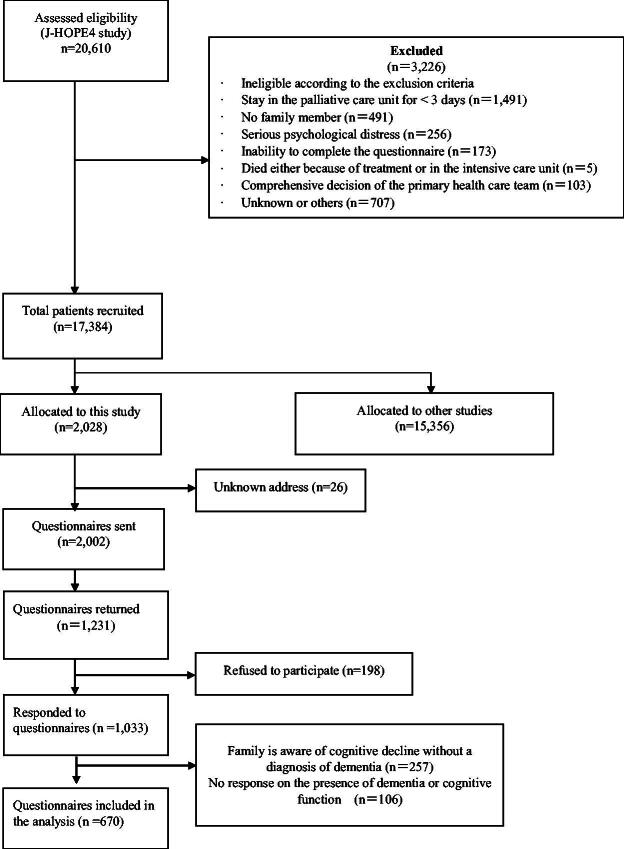
Flow diagram showing the process of selection of study subjects and the questionnaire used.

Questionnaires were sent to 2002 bereaved families, of whom 1231 (61.4%) responded (Fig. 1). We analyzed 670 (33.4%) responses, including members of 83 bereaved families of patients with coexisting cancer and dementia. The mean age of patients in the dementia comorbidity group was 83.4 ± 7.2 years; patients in the dementia comorbidity group were significantly older than those in the nondementia comorbidity group (Table 1). The mean age of the bereaved family members of patients in the dementia comorbidity group was 61.9 ± 10.0 years. The most frequent relationship with patients was sons or daughters (56.6%), with a significantly higher percentage in the dementia comorbidity group.

**Table 1.** Sociodemographic characteristics of the study participants

	Dementia comorbidity (*n* = 83)		Non-dementia comorbidity (*n* = 587)		*P*
Patients	*n*	*%*	*n*	*%*		
Age, mean (SD), years	83.4 (7.2)		75.2 (11.1)		<0.001
Gender					
Female	45	54.2	286	48.7	0.412
Male	38	45.8	301	51.3	
Days of last hospital stay, mean (SD)	43.7 (49.4)		33.2 (36.2)		0.019
Duration of anti-cancer treatment, years					
<1	50	61.7	249	42.8	0.002
>1	31	38.3	333	57.2	
Presence of nursing care certification					
Independent support level	1	1.2	63	11.2	<0.001
Care level 1	17	20.7	65	11.5	
Care level 2	22	26.8	69	12.2	
Care level 3	18	22.0	48	8.5	
Care level 4	11	13.4	28	5.0	
Care level 5	11	13.4	29	5.2	
Others/unknown	2	2.4	261	46.4	
Medical cost, JPY^a^					0.258
>100,000	57	71.3	440	77.4	
<100,000	23	28.7	128	22.6	
Primary tumor sites					
Lung	20	24.1	117	19.9	
Colon, rectum	10	12.0	72	12.3	
Head and neck	9	10.8	17	2.9	
Stomach	8	9.6	73	12.3	
Pancreas	7	8.4	84	14.3	
Prostate, kidney, bladder	5	6.0	48	8.2	
Gallbladder, bile duct	5	6.0	38	6.5	
Lymph node, blood	4	4.8	8	1.4	
Liver	3	3.6	24	4.1	
Uterus, ovary	2	2.4	29	4.9	
Breast	2	2.4	25	4.3	
Others	8	9.9	52	8.9	

Family Caregivers	*n*	%	*n*	%	
Age, mean (SD), years	61.9 (10.0)		62.8 (12.0)		0.546
Gender					
Female	58	70.7	361	62.2	0.144
Male	24	29.3	219	37.8	
Relationship with the patient					
Child	47	56.6	239	40.9	
Spouse/partner	18	21.7	256	43.8	
Child-in-law	12	14.5	20	3.4	0.001
Sibling	3	3.6	46	7.9	
Others	3	3.6	23	3.9	
Time since the patient’s death, days					
Mean (SD)	335.8 (100.2)		358.1 (134.4)		0.146
Range	189-659		160-1528		

Abbreviations: SD, standard deviation

^a^ 1 USD = 150 JPY

The total score for all 18 items of GDI - short version (dementia comorbidity vs. nondementia comorbidity, mean ± standard deviation) was 78.4 ± 17.7 versus 80.0 ± 15.5, adj *P* = 0.186, ES = 0.10 (Table 2); for the 10 items in the core domain of the GDI-short version was 46.0 ± 9.9 versus 46.9 ± 9.1, adj *P* = 0.227, ES = 0.09; for the 8 items in the additional domain of the GDI-short version was 32.4 ± 8.8 versus 33.0 ± 7.5, adj *P* = 0.205, ES = 0.08. Overall evaluation of the achievement of good death was 7.09 ± 2.40 versus 6.54 ± 2.52, adj *P* = 0.414. No statistical differences were observed in the total scores between the two groups. The highest scoring item in both groups was “being valued as a person” (6.10 ± 0.78 vs. 6.03 ± 0.86, adj *P* = 0.540, ES = 0.07). There were significant differences between the groups in the scores for eight items after adjusting for age. The scores for the following items were higher in the dementia comorbidity group: “dying without awareness that one is dying” (4.52 ± 1.64 vs. 3.37 ± 1.51, adj *P* < 0.001, ES = 0.75) and “not disclosing one’s physical and mental weakness to the family (reversal item)” (4.14 ± 1.59 vs. 3.39 ± 1.42, adj *P* < 0.001, ES = 0.51). In contrast, the items with lower scores in the dementia comorbidity group were “being able to stay at his or her favorite place” (4.51 ± 1.50 vs. 4.93 ± 1.45, adj *P* = 0.021, ES = 0.28), “feeling that one’s life is worth living” (4.37 ± 1.23 vs. 4.83 ± 1.29, adj *P* = 0.004, ES = 0.35), “saying what one wants to dear people” (3.99 ± 1.79 vs. 4.67 ± 1.54, adj *P* < 0.001, ES = 0.43), “knowing what to expect about one’s condition in the future” (3.21 ± 1.72 vs. 4.70 ± 1.49, adj *P* < 0.001, ES = 0.97), “being independent in daily activities” (2.12 ± 1.42 vs. 3.12 ± 1.83, adj *P* < 0.001, ES = 0.53), and “trusting physician” (5.31 ± 1.36 vs. 5.58 ± 1.10, adj *P* = 0.044, ES = 0.23).

**Table 2.** Achievement of Good Death in Patients with Cancer with or Without Dementia

Good Death Inventory	Dementia comorbidity (*n* = 83)		Non-dementia comorbidity (*n* = 587)				
10 core domains	Mean	SD	Mean	SD	*P*	Adj *P*	ES (*d*)
Being valued as a person	6.10	0.78	6.03	0.86	0.521	0.540	0.07
Living in calm circumstances	5.47	1.24	5.43	1.08	0.761	0.988	0.03
Being free from physical distress	5.44	1.30	5.10	1.34	0.033	0.194	0.25
Feeling that one’s life was completed	5.39	1.39	4.83	1.68	0.004	0.540	0.34
Trusting physician	5.31	1.36	5.58	1.10	0.099	0.044	0.23
Spending enough time with one’s family	5.07	1.28	5.00	1.40	0.639	0.807	0.05
Being able to stay at his or her favorite place	4.51	1.50	4.93	1.45	0.022	0.021	0.28
Having some pleasure in daily life	4.19	1.40	4.18	1.62	0.963	0.720	0.01
Not being a burden to others^a^	3.94	1.59	3.66	1.60	0.144	0.345	0.17
Being independent in daily activities	2.12	1.42	3.12	1.83	<0.001	<0.001	0.53

8 additional domains	Mean	SD	Mean	SD	*P*	Adj *P*	ES (*d*)
Dying a natural death	5.44	1.45	4.98	1.36	0.005	0.090	0.33
Receiving enough treatment	5.11	1.56	4.88	1.47	0.189	0.718	0.15
Dying without awareness that one is dying	4.52	1.64	3.37	1.51	<0.001	<0.001	0.75
Feeling that one’s life is worth living	4.37	1.23	4.83	1.29	0.003	0.004	0.35
Not exposing one’s physical and mental weakness to family^a^	4.14	1.59	3.39	1.42	<0.001	<0.001	0.51
Saying what one wants to dear people	3.99	1.79	4.67	1.54	<0.001	<0.001	0.43
Knowing what to expect about one’s condition in the future	3.21	1.72	4.70	1.49	<0.001	<0.001	0.97
Supported by religion	2.77	1.78	2.89	1.83	0.581	0.374	0.06
Total score for 10 core domains	46.06	9.93	46.98	9.18	0.400	0.227	0.09
Total score for 8 additional domains	32.40	8.88	33.00	7.50	0.456	0.205	0.08
Total score	78.47	17.78	80.06	15.57	0.394	0.186	0.10
Overall evaluation of the achievement of good death	7.09	2.40	6.54	2.52	0.070	0.414	0.21

Comparison between two groups using Student’s *t*-test, *P* < 0.05.

Score range 1–7: 1, absolutely disagree; 2, disagree; 3, somewhat disagree; 4, unsure; 5, somewhat agree; 6, agree; 7, absolutely agree. Higher score indicates the achievement of good death.

ES was standardized and calculated using Cohen’s *d*.

^a^ Inverse item.

adj, adjusted; ES, effect size.

Next, the CES2 was used to compare the EOL care structure and process between the groups (Table 3). There were no significant differences in the total score for all items after adjusting for age (49.70 ± 9.22 vs. 48.82 ± 8.40, adj *P* = 0.316, ES = 0.10).

**Table 3.** CES2 scores of patients with cancer with or without dementia

	Dementia comorbidity (*n* = 83)		Non-dementia comorbidity (*n* = 587)				
CES2	Mean	SD	Mean	SD	*P*	AdjP	ES(d)
Patient’s room is convenient and comfortable	5.37	0.82	5.07	1.02	0.013	0.011	0.29
The staff tried so that the patient’s hope could be accomplished	5.24	1.08	5.19	0.91	0.609	0.323	0.06
There is good cooperation among staff members such as physicians and nurses	5.20	0.98	4.97	1.00	0.054	0.091	0.22
Nurses had adequate knowledge and skills	5.13	1.09	5.03	0.93	0.340	0.417	0.11
Doctors had adequate knowledge and skills	5.11	1.37	5.13	0.97	0.895	0.916	0.02
Admission (use) is possible when necessary without waiting	5.02	1.11	4.88	1.23	0.307	0.629	0.12
Doctors tried to relieve physical discomfort of the patient	4.96	1.58	5.18	0.94	0.238	0.180	0.20
The total cost is reasonable	4.87	1.36	4.78	1.14	0.521	0.820	0.07
Consideration was given to the health of family	4.65	1.73	4.56	1.36	0.654	0.627	0.06
Doctors gave sufficient explanation to the patient about the expected outcome	4.51	1.88	4.54	1.52	0.884	0.892	0.02
Total score of 10 CES2 items	49.70	9.22	48.82	8.40	0.382	0.316	0.10

Comparison between two groups using student-t test, *P* < 0.05.

Abbreviations: CES2, Care Evaluation Scale2; SD, standard deviation; ES, effect size. ES was standardized and calculated using Cohen’s d.

Score range 1-6: 1, highly disagree; 2, disagree; 3, somewhat disagree; 4, somewhat agree; 5, agree; 6, highly agree. Higher score indicates better care.

Regarding the support for families by health care professionals (Table 4), statistically significant differences were observed in “explaining what families wish to know” (3.31 ± 0.64 vs. 3.12 ± 0.65, adj *P* = 0.035, ES = 0.29), “allowing sufficient time for families to think” (3.22 ± 0.69 vs. 2.89 ± 0.72, adj *P* < 0.001, ES = 0.46), “giving approval for what they choose to do” (3.08 ± 0.78 vs. 2.84 ± 0.75, adj *P* < 0.029, ES = 0.31), and “coordinating family relationship” (2.93 ± 0.84 vs. 2.694 ± 0.84, adj *P* < 0.039, ES = 0.28).

**Table 4.** Support for Family Caregivers of Patients with or Without Dementia by Health Care Professionals

	Dementia comorbidity (*n* = 83)		Non-dementia comorbidity (*n* = 587)				
	Mean	SD	Mean	SD	*P*	Adj *P*	ES (*d*)
Listening to what family caregivers would like to do	3.31	0.62	3.17	0.61	0.052	0.125	0.23
Explaining what family caregivers wish to know	3.31	0.64	3.12	0.65	0.014	0.035	0.29
Allowing sufficient time for family caregivers to think	3.22	0.69	2.89	0.72	<0.001	<0.001	0.46
Considering family caregivers’ fatigue	3.15	0.74	3.06	0.68	0.299	0.440	0.12
Giving approval for what they choose to do	3.08	0.78	2.84	0.75	0.013	0.029	0.31
Coaching family caregivers to care for patients	2.93	0.83	2.83	0.76	0.268	0.447	0.13
Coordinating family relationship	2.93	0.84	2.69	0.84	0.038	0.039	0.28

Comparison between two groups using Student’s *t*-test, *P* < 0.05.

Score range 1–4: 1, absolutely disagree; 2, disagree; 3, agree; 4, absolutely agree. Higher score indicates better care.

ES was standardized and calculated using Cohen’s *d*.

Finally, BGQ scores were lower in the dementia comorbidity group than in the nondementia comorbidity group; the difference was not significant after adjusting for age (3.40 ± 2.41 vs. 4.36 ± 2.28, adj *P* = 0.060). In addition, PHQ9 scores were lower in the dementia comorbidity group than in the nondementia comorbidity group; the difference was not significant after adjusting for age (4.67 ± 4.71 vs. 5.50 ± 5.37, adj *P* = 0.788).

## Discussion

The most significant finding of the present research was that in hospice and palliative care units, the total score for achieving good death did not differ based on comorbid dementia. From health professionals’ perspectives, dementia comorbidity negatively impacts the quality of death;^24^ however, this was not evident in our study. Meanwhile, scores for certain items were significantly lower in the dementia comorbidity group, including “saying what one wants to dear people,” “knowing what to expect about one’s condition in the future,” and “being independent in daily activities,” reflecting the decline in cognitive and physical functions. The low score for “being able to stay at his or her favorite place” could be due to the inherent difficulty in caring for a patient with both cancer and dementia at home, resulting in the family potentially not respecting the patient’s wishes. However, despite the low scores for these items, scores for “being valued as a person,” “living in calm circumstances,” and “being free from physical distress” were high in both groups, similar to previous findings.^23^ Dementia symptoms create complex and unique health care needs, rendering the provision of EOL care difficult,^9,10^ but according to the CES2 scores, good coordination among staff and health care professionals’ efforts to relieve symptoms of patients in hospice and palliative care units was evident. This may have been reflected in the GDI score. Families of patients with dementia expect that the patient’s personality is valued, they are not deprived of dignity through coercive care or communication, and they are cared for as a person.^41,42^ The quality of care for patients with dementia is reportedly higher in hospice-enrolled patients.^43^ Hospice and palliative care staff consider the pleasure and discomfort of the patient using verbal and nonverbal communication and care for their mental and physical distress. High-quality EOL care can be provided with dignity regardless of the presence of dementia, which can help achieve a good death.

Another important finding was that the support for bereaved families pertaining to several items was significantly higher in the dementia comorbidity group, whereas the degree of complicated grief and depression tended to be lower; however, the difference was not significant after adjusting for age. Mental preparation before bereavement can facilitate family adjustment after bereavement.^44^ Patients with coexisting cancer and dementia often have dementia before developing cancer, making it more difficult for patients and families to anticipate the cancer journey and be prepared for the patient’s death. Moreover, for families who expect a gradual decline in physical and cognitive functions due to dementia, the process after cancer development can be perceived as bewildering, leading to regret and a feeling of unfinished business. It is important to predict cancer progression and prognosis and pay special attention to the life story, values, preferences, and wishes of the patients and their families while considering the family’s choices. Thus, supporting patients with coexisting cancer and dementia and their families can contribute to achieving a good death and, in turn, prevent the development of complicated grief and depression in the families after bereavement.

## Study Limitations

This study had some limitations. First, it was conducted before the closure or downsizing of hospice palliative care units, reassigning of medical staff, and visitation restrictions associated with the COVID-19 pandemic. Hence, post-pandemic, the care system and staffing may differ from those pre-COVID-19 and warrant further investigation. Second, recall bias might have occurred owing to the retrospective study design. Third, we recruited participants only from hospice and palliative care units; thus, our findings may not be generalizable to other settings. Hospice care is less commonly used by patients with dementia,^45^ and many patients with severe dementia in Japan are often cared for in long-term care facilities. Therefore, achieving a good death in patients with severe dementia who die in other settings may differ from that in the study participants. Fourth, this study did not require a medical diagnosis of dementia by a physician, which may have affected the lower power of comparison between the two groups. Fifth, the questions regarding support for families by health care professionals have not been validated. Finally, a possible explanation for the lack of difference in the GDI scores between the dementia comorbidity and nondementia comorbidity groups could be that the GDI was developed for patients with cancer; however, the concept of a good death in patients with coexisting cancer and dementia may not have been adequately investigated. Hence, a unique component of a good death may exist in patients with cancer and dementia. Research is needed to address the factors affecting a good death in patients with coexisting cancer and dementia.

## Acknowledgements

We thank Japan Hospice Palliative Care Foundation and participating institutions for their cooperation.

## Conclusions

This study demonstrated no differences in achieving a good death, EOL care structure and process, and support provided by health care professionals to bereaved families owing to the presence of dementia comorbidity from the bereaved family’s perspective. Providing high-quality EOL care for patients with coexisting cancer and dementia and their families can help achieve a good death.

## Abbreviations Used

BGQ: Brief Grief Questionnaire
CES: Care Evaluation Scale
EOL: end-of-life
GDI: Good Death Inventory
PHQ: Patient Health Questionnaire

## References

[1] Fowler H, Belot A, Ellis L, et al. Comorbidity prevalence among cancer patients: A population-based cohort study of four cancers. BMC Cancer 2020;20(1):2; doi: 10.1186/s12885-019-6472-9 31987032 PMC6986047

[2] McWilliams L, Farrell C, Grande G, et al. A systematic review of the prevalence of comorbid cancer and dementia and its implications for cancer-related care. Aging Ment Health 2018;22(10):1254–1271; doi: 10.1080/13607863.2017.1348476 28718298

[3] Hanrahan P, Luchins DJ. Access to hospice programs in end-stage dementia: A national survey of hospice programs. J Am Geriatr Soc 1995;43(1):56–59; doi: 10.1111/j.1532-5415.1995.tb06243.x 7806741

[4] Aldridge MD, Hunt L, Husain M, et al. Impact of comorbid dementia on patterns of hospice use. J Palliat Med 2022;25(3):396–404; doi: 10.1089/jpm.2021.0055 34665050 PMC8968839

[5] Blytt KM, Selbæk G, Drageset J, et al. Comorbid dementia and cancer in residents of nursing homes: Secondary analyses of a cross-sectional study. Cancer Nurs 2018;41(2):E13–E20; doi: 10.1097/NCC.0000000000000478 28146014 PMC5839697

[6] Sakata N, Okumura Y, Ogawa A. Postoperative pain treatment in patients with dementia: A retrospective observational study. Drugs Aging 2022;39(4):305–311; doi: 10.1007/s40266-022-00932-3 35362866

[7] Hsu WH, Hsieh JG, Wang YW, et al. Insufficient pain control for patients with cancer and dementia during terminal cancer stages. Am J Transl Res 2021;13(11):13034–13042. 34956521 PMC8661160

[8] Kedia SK, Chavan PP, Boop SE, Yu X. Health Care Utilization Among Elderly Medicare Beneficiaries With Coexisting Dementia and Cancer. Gerontol Geriatr Med 2017;3:2333721416689042; doi: 10.1177/2333721416689042 31508440 31508440 PMC5308432

[9] Hirooka K, Okumura Y, Matsumoto S, et al. Quality of end-of-life in cancer patients with dementia: Using a nationwide inpatient database. J Pain Symptom Manage 2022;64(1):1–7; doi: 10.1016/j.jpainsymman.2022.03.016 35367609

[10] Fürst P, Strang P, Hedman C, et al. Advanced cancer and concomitant dementia: Access to specialized palliative care, emergency room, hospital care, and place of death. Acta Oncol 2022;61(7):874–880; doi: 10.1080/0284186X.2022.2062681 35411838

[11] Pivodic L, Smets T, Van den Noortgate N, et al. Quality of dying and quality of end-of-life care of nursing home residents in six countries: An epidemiological study. Palliat Med 2018;32(10):1584–1595; doi: 10.1177/0269216318800610 30273519 PMC6238165

[12] Harrison KL, Cenzer I, Smith AK, et al. Functional and clinical needs of older hospice enrollees with coexisting dementia. J Am Geriatr Soc 2023;71(3):785–798; doi: 10.1111/jgs.18130 36420734 PMC10023265

[13] Institute of Medicine (US). Committee on Care at the End of Life. Introduction. In: Approaching Death: Improving Care at the End of Life (Field MJ, Cassel CK. eds.) National Academic Press: Washington, DC; 1997. 25121204

[14] Krikorian A, Maldonado C, Pastrana T. Patient’s perspectives on the notion of a good death: A systematic review of the literature. J Pain Symptom Manage 2020;59(1):152–164; doi: 10.1016/j.jpainsymman.2019.07.033 31404643

[15] Esmaeili-Abdar M, Alborz University of Medical Sciences, Karaj, Iran. Analyzing the concepts of “good death” from the perspective of nursing: A systematic review and concept analysis. Electron Physician 2022;14(3):7898–7910.

[16] Campbell SM. Well-being and the good death. Ethical Theory Moral Pract 2020;23(3-4):607–623; doi: 10.1007/s10677-020-10101-3 32837254 PMC7357436

[17] Steinhauser KE, Clipp EC, McNeilly M, et al. In search of a good death: Observations of patients, families, and providers. Ann Intern Med 2000;132(10):825–832; doi: 10.7326/0003-4819-132-10-200005160-00011 10819707

[18] Cottrell L, Duggleby W. The “good death”: An integrative literature review. Palliat Support Care 2016;14(6):686–712; doi: 10.1017/S1478951515001285 26732508

[19] Meier EA, Gallegos JV, Thomas LP, et al. Defining a good death (successful dying): Literature review and a call for research and public dialogue. Am J Geriatr Psychiatry 2016;24(4):261–271; doi: 10.1016/j.jagp.2016.01.135 26976293 PMC4828197

[20] Miyajima K, Fujisawa D, Yoshimura K, et al. Association between quality of end-of-life care and possible complicated grief among bereaved family members. J Palliat Med 2014;17(9):1025–1031; doi: 10.1089/jpm.2013.0552 25050607

[21] Aoyama M, Sakaguchi Y, Morita T, et al. Factors associated with possible complicated grief and major depressive disorders. Psychooncology 2018;27(3):915–921; doi: 10.1002/pon.4610 29247587

[22] Hamano J, Morita T, Fukui S, et al. Trust in physicians, continuity and coordination of care, and quality of death in patients with advanced cancer. J Palliat Med 2017;20(11):1252–1259; doi: 10.1089/jpm.2017.0049 28731821

[23] Choi JY, Kong KA, Chang YJ, et al. Effect of the duration of hospice and palliative care on the quality of dying and death in patients with terminal cancer: A nationwide multicentre study. Eur J Cancer Care (Engl) 2018;27(2):e12771; doi: 10.1111/ecc.12771 28913848

[24] Hirooka K, Nakanishi M, Fukahori H, et al. Impact of dementia on quality of death among cancer patients: An observational study of home palliative care users. Geriatr Gerontol Int 2020;20(4):354–359; doi: 10.1111/ggi.13860 32020761

[25] Masukawa K, Aoyama M, Morita T, et al. The Japan hospice and palliative evaluation study 4: A cross-sectional questionnaire survey. BMC Palliat Care 2018;17(1):66; doi: 10.1186/s12904-018-0319-z 29678173 PMC5909241

[26] Takao A, Arao H, Yamamoto S, et al. Nationwide survey on caregiver burden when supporting terminal cancer patients with dementia: Bereaved family members’ perspective. J Palliat Care 2023;38(3):326–335; doi: 10.1177/08258597231169625 37066441

[27] Abe M, Tsunawaki S, Matsuda M, et al. Perspectives on disclosure of the dementia diagnosis among primary care physicians in Japan: A qualitatively driven mixed methods study. BMC Fam Pract 2019;20(1):69; doi: 10.1186/s12875-019-0964-1 31122197 PMC6533714

[28] Iijima Y. A survey of informed consent in patients with dementia in the US and Japan. Nagoya J Med Sci 2023;85(4):797–806; doi: 10.18999/nagjms.85.4.797 38155614 PMC10751487

[29] Miyashita M, Morita T, Sato K, et al. Good death inventory: A measure for evaluating good death from the bereaved family member’s perspective. J Pain Symptom Manage 2008;35(5):486–498; doi: 10.1016/j.jpainsymman.2007.07.009 18358685

[30] Mori M, Kuwama Y, Ashikaga T, et al. Acculturation and perceptions of a good death among Japanese Americans and Japanese living in the U.S. J Pain Symptom Manage 2018;55(1):31–38; doi: 10.1016/j.jpainsymman.2017.08.010 28842219

[31] Roshan HM, Ebadi A, Karimi L, et al. Translation and psychometric evaluation of the Persian version of “good death inventory-short Form” from the perspective of family-members of cancer patients. BMC Psychol 2023;11(1):261; doi: 10.1186/s40359-023-01305-0 37660187 PMC10475178

[32] Miyashita M, Morita T, Tsuneto S, et al. The Japan hospice and palliative care evaluation study (J-HOPE study): Study design and characteristics of participating institutions. Am J Hosp Palliat Care 2008;25(3):223–232; doi: 10.1177/1049909108315517 18573997

[33] Aoyama M, Morita T, Kizawa Y, et al. The Japan hospice and palliative care evaluation study 3: Study design, characteristics of participants and participating institutions, and response rates. Am J Hosp Palliat Care 2017;34(7):654–664; doi: 10.1177/1049909116646336 27141016

[34] Miyashita M, Aoyama M, Nakahata M, et al. Development the care evaluation scale version 2.0: A modified version of a measure for bereaved family members to evaluate the structure and process of palliative care for cancer patient. BMC Palliat Care 2017;16(1):8; doi: 10.1186/s12904-017-0183-2 28114917 PMC5259912

[35] Courtier N, Milton R, King A, et al. Cancer and dementia: An exploratory study of the experience of cancer treatment in people with dementia. Psychooncology 2016;25(9):1079–1084; doi: 10.1002/pon.4212 27423160

[36] Moore KJ, Davis S, Gola A, et al. Experiences of end of life amongst family carers of people with advanced dementia: Longitudinal cohort study with mixed methods. BMC Geriatr 2017;17(1):135; doi: 10.1186/s12877-017-0523-3 28673257 PMC5496359

[37] Shear KM, Jackson CT, Essock SM, et al. Screening for complicated grief among Project Liberty service recipients 18 months after September 11, 2001. Psychiatr Serv 2006;57(9):1291–1297; doi: 10.1176/ps.2006.57.9.1291 16968758

[38] Löwe B, Kroenke K, Herzog W, et al. Measuring depression outcome with a brief self-report instrument: Sensitivity to change of the Patient Health Questionnaire (PHQ-9). J Affect Disord 2004;81(1):61–66; doi: 10.1016/S0165-0327(03)00198-8 15183601

[39] Ito M, Nakajima S, Fujisawa D, et al. Brief measure for screening complicated grief: Reliability and discriminant validity. PLoS One 2012;7(2):e31209; doi: 10.1371/journal.pone.0031209 22348057 PMC3279351

[40] Muramatsu K, Miyaoka H, Kamijima K, et al. Performance of the Japanese version of the patient health questionnaire-9 (J-PHQ-9) for depression in primary care. Gen Hosp Psychiatry 2018;52:64–69; doi: 10.1016/j.genhosppsych.2018.03.007 29698880

[41] Nishimura M, Kohno A, van der Steen JT, et al. Conceptualization of a good end-of-life experience with dementia in Japan: A qualitative study. Int Psychogeriatr 2020;32(2):255–265; doi: 10.1017/S1041610219001017 31455444

[42] Nishimura M, Dening KH, Sampson EL, et al. Cross-cultural conceptualization of a good end of life with dementia: A qualitative study. BMC Palliat Care 2022;21(1):106; doi: 10.1186/s12904-022-00982-9 35676673 PMC9175529

[43] Harrison KL, Cenzer I, Ankuda CK, et al. Hospice improves care quality for older adults with dementia in their last month of life. Health Aff (Millwood) 2022;41(6):821–830; doi: 10.1377/hlthaff.2021.01985 35666964 PMC9662595

[44] Wen F-H, Chou W-C, Hou M-M, et al. Associations of death-preparedness states with bereavement outcomes for family caregivers of terminally ill cancer patients. Psychooncology 2022;31(3):450–459; doi: 10.1002/pon.5827 34549848

[45] Xiong B, Freeman S, Banner D, et al. Hospice utilization among residents in long-term care facilities. J Palliat Care 2021;36(1):50–60; doi: 10.1177/0825859720907415 32093589

